# Scarlet Fever and Disease in Cattle

**Published:** 1888-04

**Authors:** William Osler


### EDITORIAL DEPARTMENT.

### SCARLET FEVER AND DISEASE IN CATTLE.

BY WILLIAM OSLER, M. D.

In London in 1885–86 there was an outbreak of scarlet fever in the Marylebone district which Dr. Klein traced, he thought, in connection with a disease among the cows on a dairy farm at Hendon. Investigation showed this so-called Hendon disease to be a specific contagious affection communicable to cows and also by inoculation to man. Dr. Klein believed it to be closely related to scarlatina, and suggested that the disease was induced by drinking the milk of infected animals. The disease in the cows was characterized by enlargement of the teats and an eruption of vesicles or bullæ, ranging in size from a pea to a bean. When the vesicles were rubbed off in milking, raw sores were left, on which brown scabs formed. The vescicles and ulcers were sometimes seen on the udder. The affected animals were thin, but fed well and gave full quantities of milk. In some cases there were initial symptoms, such as cough, fever, sore throat and discharges from the eyes and nostrils. It was believed to be a disease distinct from cow-pox. Dr. Klein succeeded in isolating a micrococcus with culture of which he inoculated calves and produced a disease closely resembling scarlet fever. In human scarlet fever Dr. Klein found a micrococcus in four out of eleven cases, which appeared to be identical in its mode of growth with the organism found in the Hendon cow disease, and when inoculated in calves produced a similar infection. He concluded "that the danger of scarlatinal infection from the disease in the cow is real and that towards the study and careful supervision of this cow disease all efforts ought to be directed to check the spread of scarlet fever in man." The subject has been recently studied by Crookshank (under the direction of the agricultural department of the Privy Council), who has had an opportunity of seeing two outbreaks, similar apparently to the Hendon disease. In the first there was a characteristic eruption on the udder and teats, sores covered with crusts, and from the bases of these, cultures were made. Inoculation in calves direct from the discharge resulted in the production of vesicles and crusts which thickened gradually and reached a height on the eighth day. The cultures showed a coccus identical apparently with that described by Klein. Inspection of an infected herd gave most interesting results. The disease had spread through about 160 cows. The milkers stated that the teats swelled, were hot to the touch, and very tender so that the animals were milked with difficulty. They also stated that a little white bladder would rise on the teat, reach the size of a pea and then burst or be broken, and upon the sore a scab would form. Crookshank found that the disease had been communicated to several of the men who had sores on the hands very similar to those on the teats of the cow, with crusts of an identical character. He had an opportunity of seeing a recent case which conformed in every respect with ordinary vaccinia and from the lymph of a typical vesicle, calves were inoculated with the production of ordinary cow-pox. He claims, therefore, that the Hendon disease and the infection which he studied, are examples of spontaneous cow-pox, and have nothing whatever, to do with scarlatina. Certainly the development of a typical pox as the result of accidental inoculation and the production of true vaccinia in the calf with the lymph, seems to fulfill the conditions necessary for proving that the disease was true cow-pox. The outbreak which Crookshank investigated was apparently identical with the Hendon disease. We shall doubtless, however, hear from Dr. Klein on this point. It is well to bear in mind that there is more than one vesicular disease of the udder and teats of the cow. Jenner described a spurious cow-pox inoculation which gave no protection against small-pox, and Ceely distinguished three varieties of the spurious pox.

Apart from the value of Crookshank's observations in thus elucidating the true nature of this much talked of Henden cow disease, the discovery of the prevalence of cowpox in England is of very great interest. It was believed to have disappeared and the vaccine establishments had to send to France and Belgium for a supply of calf lymph to start the stock.

A curious attempt has been made by Dr. Stickler, of Orange, N. J., (Medical Record, December 10th, 1887,) to show a relationship between foot and mouth disease and human scarlet fever. At Dover, England, in 1884, a large number of people had sore throat which was believed to be due to drinking milk from cows affected with foot and mouth disease. Sore throat and inflammation of the cervical glands were the prominent symptoms. Two years subsequent to the epidemic Dr. Stickler gathered statistics from 183 persons who had had the sore throat and found that none of them had subsequently had scarlet fever. In twenty-four cases the throat epidemic failed to attack those who had had scarlatina. He also inoculated three children with virus from foot and mouth disease. who on subsequent exposure, did not take scarlet fever. These results, he says, "*seem* to furnish some reason for believing that, for a time at least, the virus of foot and mouth disease is protective against the contagium of scarlet fever."

The subject is one deserving of careful study and it is quite possible that from one of the lower animals we may obtain a virus as harmless as vaccinia and at the same time protective against one of the epidemic diseases of man.

